# Commentary: Enhanced Metabolic Stress Augments Ischemic Preconditioning for Exercise Performance

**DOI:** 10.3389/fphys.2019.01388

**Published:** 2019-11-13

**Authors:** Moacir Marocolo, Anderson Meireles, Gustavo R. da Mota

**Affiliations:** ^1^Physiology and Human Performance Research Group, Department of Physiology, Federal University of Juiz de Fora, Juiz de Fora, Brazil; ^2^Human Performance and Sports Research Group, Department of Sport Sciences, Institute of Health Sciences, Federal University of Triangulo Mineiro, Uberaba, Brazil

**Keywords:** exercise, blood flow occlusion, ischemic precondioning, electrical stimualtion, sports, skeletal muscle

Our research group has published several papers involving ischemic preconditioning (IPC) and exercise performance (Marocolo et al., 2017, 2018, 2019). Thus, we read with interest the paper of Slysz and Burr (2018). The aim of their study was to identify the combined effect of increasing tissue level oxygen consumption and metabolite accumulation on the ergogenic efficacy of ischemic preconditioning (IPC) during maximal aerobic and maximal anaerobic exercise. Briefly, the authors concluded that IPC combined with walking or electrical muscle stimulation (EMS) improved performance in a maximal aerobic test to exhaustion, but traditional (i.e., isolated) IPC no. Also, they found no effects from all treatments on maximal oxygen consumption and maximal anaerobic power (Slysz and Burr, 2018). Although their article is relevant and feasible, we would like to promote intellectual discussion aiming to full the topic. Therefore, we present some concerns on the validity of their conclusions.

1. The authors did not have “controls/placebo” for EMS and walking isolated in order to exclude a potential beneficial effect from EMS/walking “only” (i.e., no IPC). In this case, the authors should test the same hypothesis but with one trial performing placebo or SHAM (low pressure cuff) + EMS, and another trial performing SHAM (low pressure cuff) + walking (i.e., both no “real” IPC). On the current way, the authors cannot guarantee that amplification of the IPC stimulus augments the effect for exercise capacity since they did not test both the walking protocol and the EMS with a SHAM/placebo. In other words, would be the beneficial results from IPC, or only due to EMS or walking effects? It seems logical that only EMS (or walking) can increase tissue level oxygen consumption, blood flow and metabolite accumulation (Levine et al., 1990; Muthalib et al., 2010, 2016). Thus, we believe that their conclusion is not supported by the data.
2. Additionally, it is unclear if the authors kept blinded the evaluators of the physical tests from previous conditions (e.g., IPC, IPC + EMS). Since some studies have described a possible motivational effect due cuff intervention (Marocolo et al., 2015, 2016; De Souza et al., 2019), a previous knowledge from which intervention was carried out prior the tests could be a significant fact to alter their results (Slysz and Burr, 2018).
3. The authors did not provide rationale for the walking protocol. The reader should be aware that the same treadmill speed (i.e., 2 mph) for all subjects, may have leaded to different metabolic stress on different participants (e.g., VO_2max_ range from ~40 to 55 mL.kg.min^−1^). Unfortunately, the authors did not present any physiological indicator (e.g., heart rate or blood lactate response) from the walking protocol. Additionally, maybe the precise term would be not “IPC,” but “walking exercise with restricted leg muscle blood flow” as the authors cited in their reference list (Sakamaki et al., 2011). IPC is characterized by total blood flow occlusion alternating with reperfusion at rest, and before the exercise test. On the other hand, exercise with restricted muscle blood flow is performing “during” the exercise (i.e., not preconditioning). These two different methods, *per si*, may result in different physiological responses.
4. In the discussion, the authors stated: “*The effect of an 11–15 W increase in max power could be quite meaningful in a competition situation…*” We have doubts about the level of the subjects (VO_2max_ range from ~40 to 55 mL.kg.min^−1^) to mention that 11–15 W of increment would be relevant in a competition. Due to the fitness level of their participants, probably the enhancement from IPC, combined with walking or EMS, is not relevant in practical terms (Marocolo et al., 2018, 2019).

## Author Contributions

MM and GM contributed to the conception and designing of the study. AM, MM, and GM contributed to the revision and edition of the manuscript. All of the authors approved the final version of the manuscript.

### Conflict of Interest

The authors declare that the research was conducted in the absence of any commercial or financial relationships that could be construed as a potential conflict of interest.
